# Vitamin D_3_ suppresses Npt2c abundance and differentially modulates phosphate and calcium homeostasis in Npt2a knockout mice

**DOI:** 10.1038/s41598-024-67839-4

**Published:** 2024-07-23

**Authors:** Linto Thomas, Lashodya V. Dissanayake, Maryam Tahmasbi, Alexander Staruschenko, Sima Al-Masri, Jessica A. Dominguez Rieg, Timo Rieg

**Affiliations:** 1 Department of Molecular Pharmacology and Physiology, Morsani College of Medicine, University of South Florida, Tampa, FL USA; 2 James A. Haley Veterans’ Hospital, Tampa, FL USA; 3 Department of Pathology and Cell Biology, University of South Florida, Tampa, FL USA; 4 Hypertension and Kidney Research Center, University of South Florida, Tampa, FL USA

**Keywords:** Calcium, Fibroblast growth factor 23, Parathyroid hormone, Sodium-phosphate cotransporter, Vitamin D_3_, Calcium and vitamin D, Renal calculi

## Abstract

Vitamin D_3_ is clinically used for the treatment of vitamin D_3_ deficiency or osteoporosis, partially because of its role in regulating phosphate (P_i_) and calcium (Ca^2+^) homeostasis. The renal sodium-phosphate cotransporter 2a (Npt2a) plays an important role in P_i_ homeostasis; however, the role of vitamin D_3_ in hypophosphatemia has never been investigated. We administered vehicle or vitamin D_3_ to wild-type (WT) mice or hypophosphatemic Npt2a^−/−^ mice. In contrast to WT mice, vitamin D_3_ treatment increased plasma P_i_ levels in Npt2a^−/−^ mice, despite similar levels of reduced parathyroid hormone and increased fibroblast growth factor 23. Plasma Ca^2+^ was increased ~ twofold in both genotypes. Whereas WT mice were able to increase urinary P_i_ and Ca^2+^/creatinine ratios, in Npt2a^−/−^ mice, P_i_/creatinine was unchanged and Ca^2+^/creatinine drastically decreased, coinciding with the highest kidney Ca^2+^ content, highest plasma creatinine, and greatest amount of nephrocalcinosis. In Npt2a^−/−^ mice, vitamin D_3_ treatment completely diminished Npt2c abundance, so that mice resembled Npt2a/c double knockout mice. Abundance of intestinal Npt2b and claudin-3 (tight junctions protein) were reduced in Npt2a^−/−^ only, the latter might facilitate the increase in plasma P_i_ in Npt2a^−/−^ mice. Npt2a might function as regulator between renal Ca^2+^ excretion and reabsorption in response to vitamin D_3_.

## Introduction

Active vitamin D_3_ or 1,25-dihydroxyvitamin D_3_ (1,25(OH)_2_D_3_) is produced via the combined actions of skin, liver and kidneys^1^. Under normal conditions, it plays an essential role in the regulation of calcium (Ca^2+^) and phosphate (P_i_) homeostasis in the body^2^. A lack of vitamin D_3_ can lead to rickets and other potential processes beyond bone health, including immune system dysregulation, development of cancer, or progression of cardiovascular disease^3^. The actions of vitamin D_3_ are complex and involve hormones such as parathyroid hormone (PTH) and fibroblast growth factor 23 (FGF23)^4–6^. PTH and FGF23 are both phosphaturic hormones (via action on the renal Na^+^-P_i_ transporters, Npt2a and Npt2c) which work collaboratively to maintain P_i_ homeostasis; however, this process involves a complicated regulatory role of 1,25(OH)_2_D_3_^4,7^. 1,25(OH)_2_D_3_ has opposing effects on these hormones: it enhances the production of FGF23 in bone, while simultaneously suppressing the synthesis of PTH^8,9^. In this complex feedback loop, where PTH and FGF23 normally promote renal P_i_ excretion, 1,25(OH)_2_D_3_ may act as a switch between P_i_ excretion/absorption in order to maintain total body P_i_^4^. The precursor of vitamin D_3_, previtamin D_3_, is formed by the skin and subsequently undergoes spontaneous isomerization to vitamin D_3_, which has a half-life of ~ 26 h^10^. Any excess vitamin D_3_ is stored mainly within fat tissue^11^. In the liver, an initial hydroxylation step takes place which converts vitamin D_3_ into 25(OH) vitamin D_3_. After reaching the kidneys via the circulation, 25(OH) vitamin D_3_ undergoes a second hydroxylation process to form 1,25(OH)_2_D_3_^4,7^. PTH is part of a feedback loop that facilitates the production of 1,25(OH)_2_D_3_ by inducing the transcription of renal 1α-hydroxylase, the enzyme responsible for this process^4^. Hormonal signaling of 1,25(OH)_2_D_3_ is mediated by activation of the vitamin D receptor (VDR)^12^ and many of its actions were described by studying VDR knockout (VDR^−/−^) mice^13^. When VDR^−/−^ mice mature on a control diet, they develop rickets because of hypophosphatemia, hypocalcemia, elevated plasma 1,25(OH)_2_D_3_ and PTH levels^14^. This was found mainly to be the consequence of impaired intestinal P_i_ absorption rather than a renal P_i_ problem. Vice versa, administering 1,25(OH)_2_D_3_ to wild-type mice (WT) led to enhanced intestinal P_i_ absorption^15^, an effect absent in mice lacking the intestinal Na^+^-P_i_ transporter (Npt2b)^15^ implying a direct effect on transcellular P_i_ transport.

The importance of Npt2a for renal P_i_ reabsorption has been demonstrated in Npt2a knockout (Npt2a^−/−^) mice. These mice are characterized by renal P_i_ wasting consequently leading to hypophosphatemia, hypoparathyroidism, reduced FGF23 levels and hypercalcemia^16–18^. Of note, effects of Npt2a knockout on plasma 1,25(OH)2D_3_ have shown to be increased^16,18^ or unchanged^4^. This finding might be related to the age when mice are studied because 1,25(OH)2D_3_ levels decreased over time in Npt2a^−/−^ mice between day 8 to day 35^19^.

Despite these known factors, the effects of vitamin D_3_ on P_i_ and Ca^2+^ regulation are incompletely understood, especially in the context of renal Npt2a and the involved complex regulatory pathways involving PTH and FGF23. In order to address this question, we treated mice lacking Npt2a (Npt2a^−/−^) with vitamin D_3_ and studied the impact on P_i_ and Ca^2+^ homeostasis. Our results demonstrate that vitamin D_3_ plays a distinct role in regulating P_i_ homeostasis, Ca^2+^ balance, and suggest the existence of novel regulatory pathways involving functional Npt2a for the regulation of P_i_ homeostasis.

## Results

### Lower plasma P_i_ and a greater increase of plasma P_i_ in response to vitamin D_3_ treatment in Npt2a^−/−^ compared to WT mice

**Figure 1:**
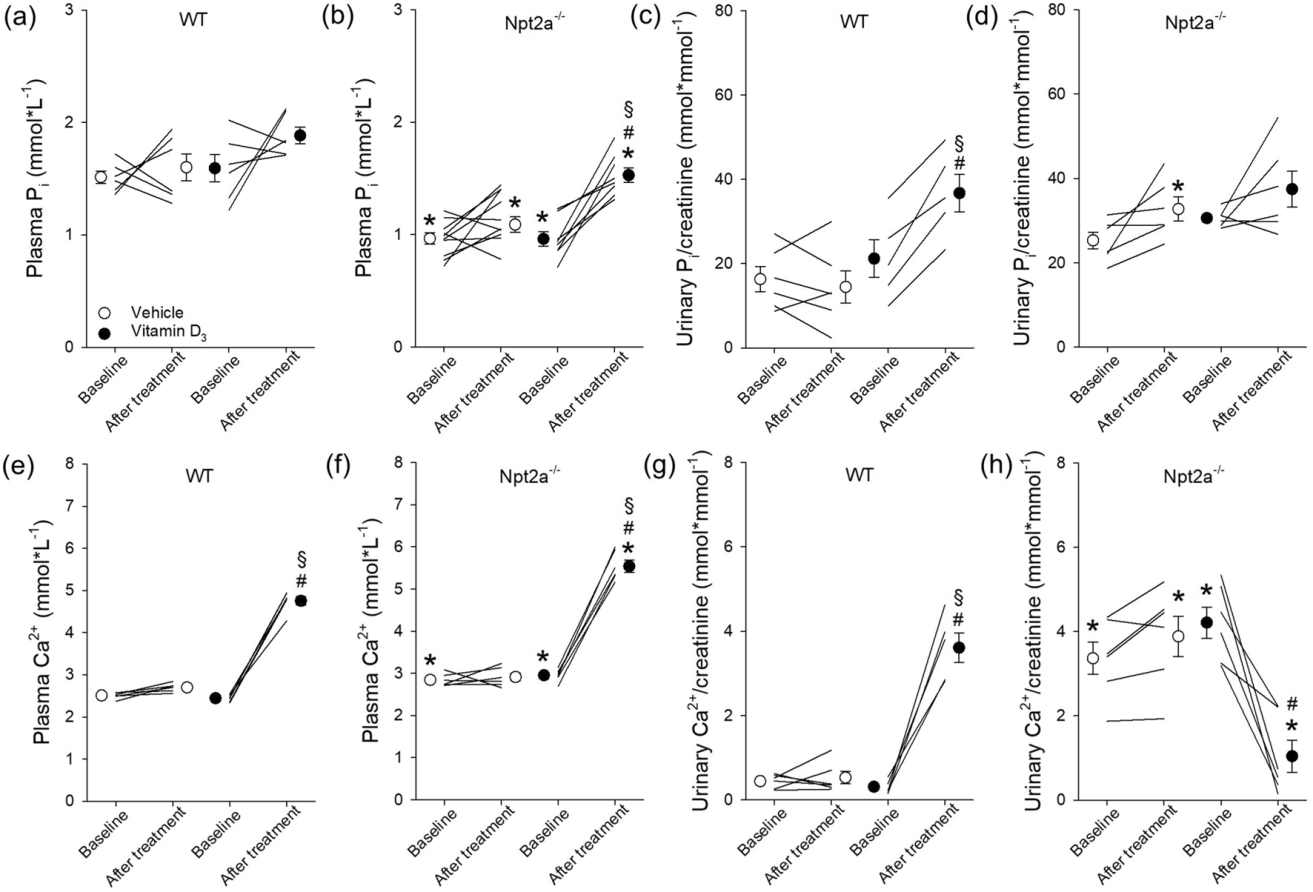
Lack of Npt2a unravels a link of vitamin D_3_ on plasma P_i_. Measurements of plasma and urinary P_i_ and Ca^2+^ were conducted in WT and Npt2a^−/−^ mice after 4 days of treatment with either a vehicle or vitamin D_3_ (n = 6–10 per genotype). (**a**) In WT mice, plasma P_i_ levels remained unchanged following vitamin D_3_ treatment. (B) In contrast, lower plasma P_i_ levels under baseline conditions in Npt2a^−/−^ mice significantly increased in response to vitamin D_3_ treatment. (**c**) The urinary P_i_/creatinine ratio in WT mice increased significantly in response to vitamin D_3_ treatment. (**d**) This ratio in Npt2a^−/−^ mice was unchanged (**d**). Plasma Ca^2+^ levels in both WT and Npt2a^−/−^ mice showed a significant increase following vitamin D_3_ treatment (**e** & **f**). In WT mice, the urinary Ca^2+^ to creatinine ratio significantly increased after vitamin D_3_ treatment (**g**). In contrast, this ratio significantly decreased in Npt2a^−/−^ mice (**h**). Male mice were used in these studies. In addition to single data summary data are shown and are expressed as mean ± SEM and were analyzed by repeated-measures two-way ANOVA followed by Tukey’s multiple comparisons test. *P < 0.05 vs WT same time point, ^*#*^*P* < 0.05 vs baseline same genotype, ^§^*P* < 0.05 vs vehicle same genotype and time point.

Consistent with previous reports^16,20^, plasma P_i_ in Npt2a^−/−^ mice was significantly lower compared to WT mice (Fig. 1a,b). In WT mice, neither vehicle treatment nor vitamin D_3_ (300,000 IU/kg body weight) treatment affected plasma P_i_ levels (Fig. 1a). In contrast, plasma P_i_ significantly increased in Npt2a^−/−^ mice (~ 1.6-fold), whereas vehicle treatment was without effect (Fig. 1b). Under baseline conditions, urinary P_i_/creatinine ratios were not significantly different between genotypes (Fig. 1c,d). In WT mice, the urinary P_i_/creatinine ratio remained unaltered in response to vehicle treatment but vitamin D_3_ treatment resulted in a significant increase (~ 1.7-fold) compared to baseline (Fig. 1c). No significant changes were observed in urinary P_i_/creatinine ratios in response to vehicle or vitamin D_3_ treatment in Npt2a^−/−^ mice (Fig. 1d).

Npt2a^−/−^ mice showed significantly higher plasma Ca^2+^ levels (~ 1.2-fold) compared to WT mice (Fig. 1e,f). Vehicle treatment did not affect plasma Ca^2+^ levels (Fig. 1e,f) in WT or Npt2a^−/−^ mice. Vitamin D_3_ treatment significantly increased plasma Ca^2+^ in WT (~ 2.0-fold) and Npt2a^−/−^ (~ 1.8-fold) mice. Of note, plasma Ca^2+^ was significantly greater in Npt2a^−/−^ mice compared to WT mice in response to vitamin D_3_ treatment (Fig. 1e,f). Under baseline conditions, urinary Ca^2+^/creatinine ratios were significantly greater in Npt2a^−/−^ compared to WT mice (Fig. 1g,h). In WT mice, the urinary Ca^2+^/creatinine ratio remained unaltered in response to vehicle treatment; however, consistent with increased plasma Ca^2+^ in response to vitamin D_3_ treatment, the urinary Ca^2+^/creatinine ratio was appropriately increased (~ 11-fold). In contrast to WT mice, the urinary Ca^2+^/creatinine ratio in Npt2a^−/−^ mice was significantly decreased (~ 50%) in response to vitamin D_3_ treatment; vehicle treatment was without effect (Fig. 1h).

Using a smaller dose of vitamin D_3_ (3000 IU/kg body weight) showed no significant effects on plasma P_i_ or urinary P_i_/creatinine ratios between genotypes (Supplementary Fig. 1a,b). Whereas plasma Ca^2+^ and urinary Ca^2+^/creatinine ratios were not affected by vitamin D_3_ in WT mice (Supplementary Fig. 1c,d), in Npt2a^−/−^ mice, plasma Ca^2+^ and urinary Ca^2+^/creatinine ratios significantly increased (~ 1.05-fold and ~ 2.7-fold, respectively).

### Npt2a^−/−^ mice lack PTH responses but FGF23 levels were significantly increased in response to vitamin D_3_ treatment

**Figure 2:**
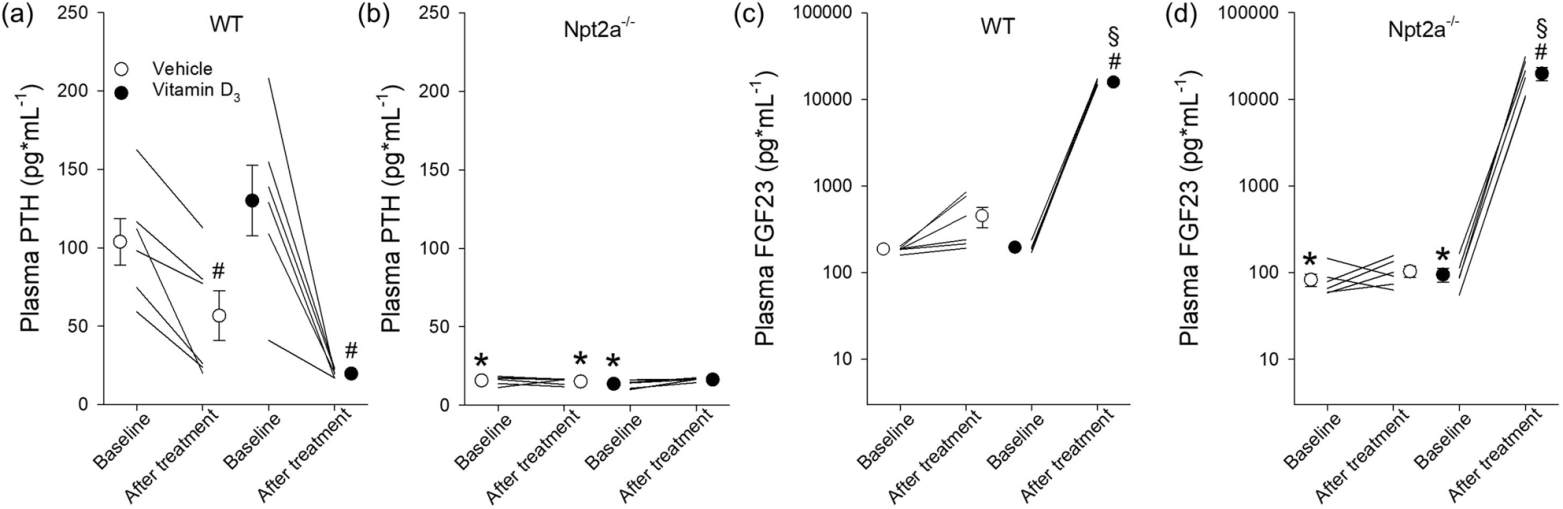
Vitamin D_3_ induces divergent responses in plasma PTH and increases FGF23 in both WT or Npt2a^−/−^ mice. Measurements of plasma PTH and FGF23 were performed in WT and Npt2a^−/−^ mice following 4 days of treatment with either vehicle or vitamin D_3_ (n = 6 per genotype). (**a**) In WT mice, vitamin D_3_ treatment led to a decrease in plasma PTH levels. (**b**) In Npt2a^−/−^ mice, plasma PTH levels were lower and unchanged in response to vitamin D_3_ treatment. (**c** & **d**) FGF23 levels significantly increased in both genotypes in response to vitamin D_3_ treatment. Male mice were used in these studies. In addition to single data summary data are shown and are expressed as mean ± SEM and were analyzed by repeated-measures two-way ANOVA followed by Tukey’s multiple comparisons test. *P < 0.05 vs WT same time point, ^*#*^*P* < 0.05 vs baseline same genotype, ^§^*P* < 0.05 vs vehicle same genotype and time point.

Npt2a^−/−^ mice show significantly lower plasma PTH levels under baseline conditions (Fig. 2a,b). In WT mice, plasma PTH showed a small but significant decrease in response to vehicle treatment (Fig. 2a). Vitamin D_3_ treatment significantly decreased (~ 85%) plasma PTH (Fig. 2a) in WT mice. No significant changes in plasma PTH were observed in response to vehicle or vitamin D_3_ treatment in Npt2a^−/−^ mice (Fig. 2b). In addition to lower PTH levels in Npt2a^−/−^ mice under baseline conditions, FGF23 levels were also significantly lower (~ 50%) compared to WT mice (Fig. 2c,d). Vehicle treatment did not significantly change FGF23 levels in either genotype (Fig. 2c,d). Vitamin D_3_ treatment caused a significant increase of FGF23 levels in both genotypes: in WT mice an ~ 80-fold increase was observed, whereas in Npt2a^−/−^ mice a ~ 200-fold increase was observed. The more than double increase of FGF23 in Npt2a^−/−^ compared to WT mice in response to vitamin D_3_ treatment is the consequence of the significantly lower baseline levels because FGF23 levels were not significantly different in response to vitamin D_3_ treatment between genotypes.

### Vitamin D_3_ has distinct effects on bone remodeling markers in WT and Npt2a^−/−^ mice

**Figure 3:**
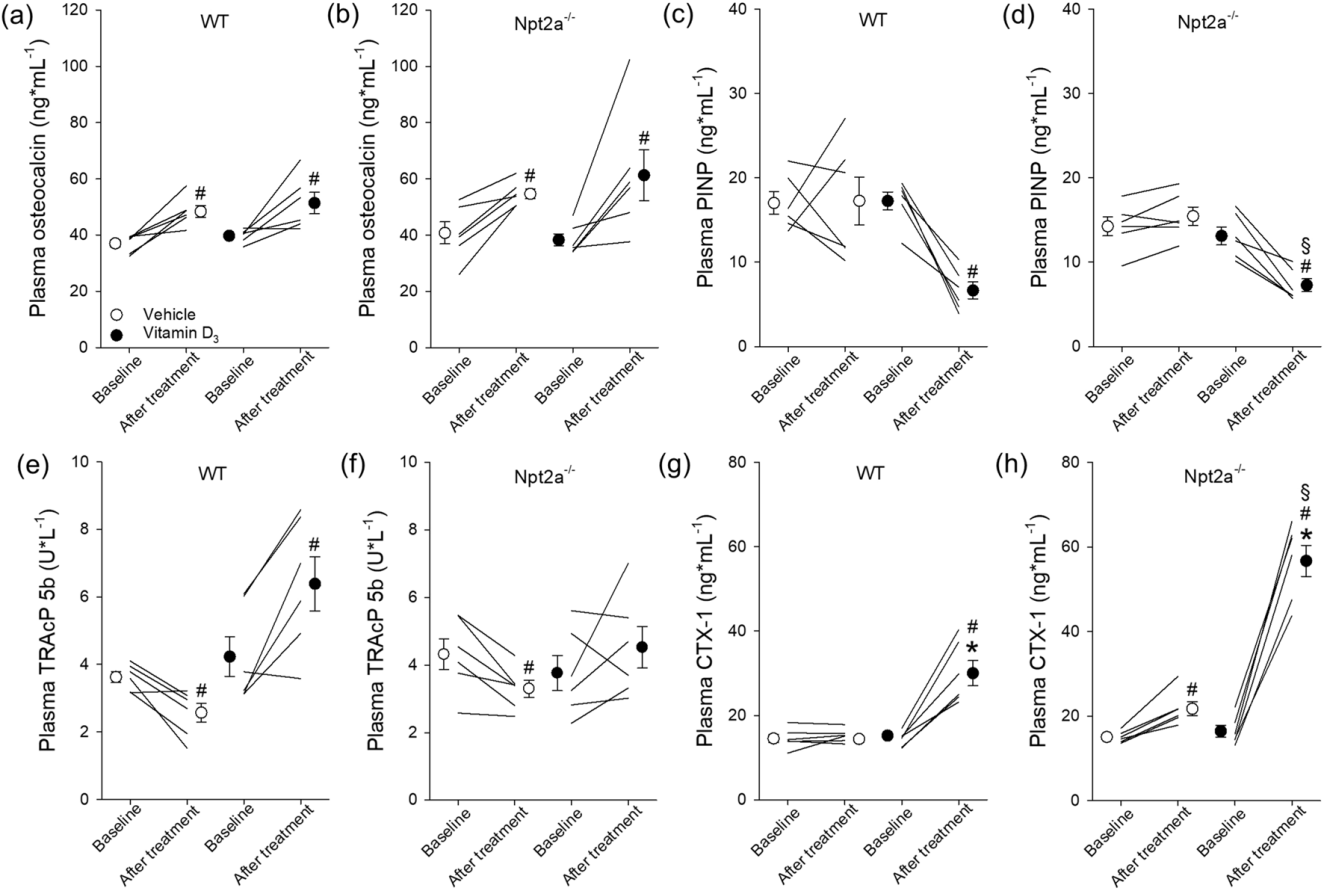
Npt2a determines the effects of Vitamin D_3_ on bone remodeling markers. Circulating bone markers, including osteocalcin, PINP, TRAcP 5b, and CTX-1 were measured in WT and Npt2a^−/−^ mice after 4 days of treatment with either a vehicle or vitamin D_3_ (n = 6 per genotype). (**a** & **b**) Both genotypes show a small but significant increase in osteocalcin levels independent of treatment. (**c** & **d**) Vitamin D_3_ decreased plasma PINP independent of genotype. (**e** & **f**) In both genotypes, vehicle treatment slightly but significantly decreased plasma TRAcP 5b levels but vitamin D_3_ only significantly increased TRAcP 5b in WT mice. (**g** & **h**) Vitamin D_3_ treatment increased CTX-1 levels in both genotypes but to a significantly greater extent in Npt2a^−/−^ mice. Male mice were used in these studies. In addition to single data summary data are shown and are expressed as mean ± SEM and were analyzed by repeated-measures two-way ANOVA followed by Tukey’s multiple comparisons test. *P < 0.05 vs WT same time point, ^*#*^*P* < 0.05 vs baseline same genotype, ^§^*P* < 0.05 vs vehicle same genotype and time point.

Since vitamin D_3_ is instrumental for bone remodeling, we determined 2 bone formation markers (osteocalcin and PINP) and 2 bone resorption markers (TRAcP 5b and CTX-1) under baseline conditions and in response to vehicle or vitamin D_3_ treatment. Under baseline conditions, no significant differences were observed in osteocalcin levels between genotypes, and vehicle and vitamin D_3_ treatment resulted in a small but significant increase in osteocalcin levels independent of genotype (Fig. 3a,b). Plasma PINP levels were not significantly different between genotypes under baseline conditions, and vehicle treatment did not significantly change PINP levels in either genotype (Fig. 3c,d). Vitamin D_3_ treatment significantly reduced (~ 60%) PINP levels in WT mice, and a similar reduction (~ 44%) was observed in Npt2a^−/−^ mice. Plasma TRAcP 5b levels were not significantly different between genotypes under baseline conditions, and in both genotypes TRAcP 5b levels slightly but significantly decreased in response to vehicle treatment. Vitamin D_3_ treatment significantly increased (~ 1.5-fold) TRAcP 5b levels in WT mice but was without significant effect in Npt2a^−/−^ mice (Fig. 3e,f). Plasma CTX-1 levels were not significantly different between genotypes under baseline conditions, and vehicle treatment did not significantly affect CTX-1 levels in either genotype (Fig. 3g,h). Vitamin D_3_ treatment significantly increased (~ twofold) CTX-1 levels in WT mice and even further increased (~ 3.5-fold) CTX-1 in Npt2a^−/−^ mice. Consequently, CTX-1 levels were significantly higher in Npt2a^−/−^ compared to WT mice after vitamin D_3_ treatment (Fig. 3g,h).

### Acute oral P_i_ loading results in greater plasma P_i_ levels in response to vitamin D_3_ treatment compared to vehicle

**Figure 4:**
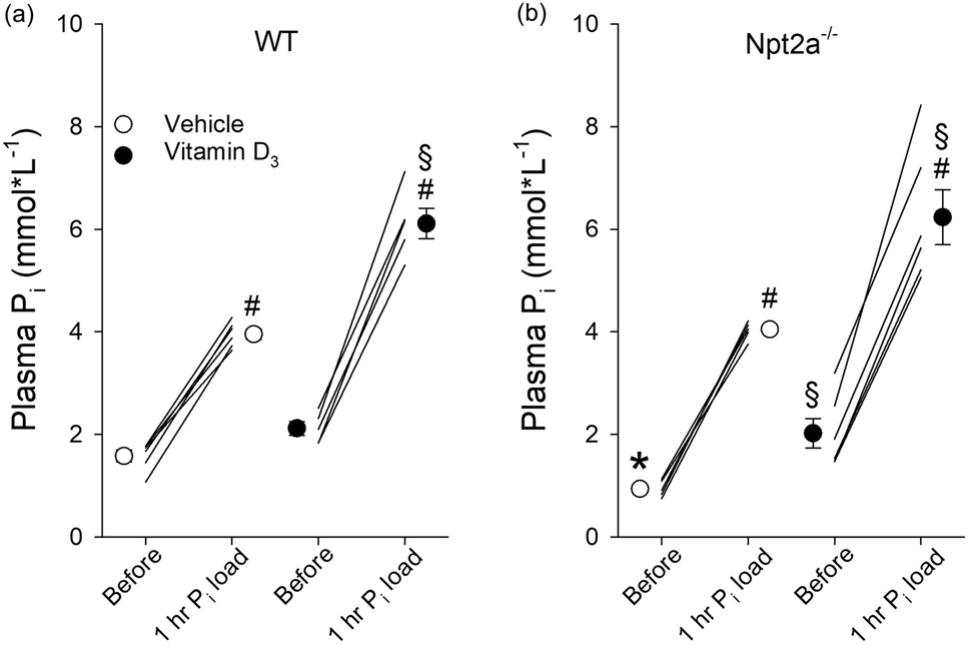
Similar effects of acute oral P_i_ loading on plasma P_i_ levels between genotypes. Plasma P_i_ levels were measured in WT and Npt2a^−/−^ mice before and one hour after oral P_i_ loading via gavage (0.5 mol*L^−1^, 1% of body weight). These measurements were performed following 4 days of treatment with either a vehicle or vitamin D_3_ (n = 6 per genotype). (**a** & **b**) Oral P_i_ loading significantly increased plasma P_i_ levels independent of genotype or treatment; however, in vitamin D_3_-treated mice the increase was significantly greater compared to vehicle-treated mice. Male mice were used in these studies. In addition to single data summary data are shown and are expressed as mean ± SEM and were analyzed by repeated-measures two-way ANOVA followed by Tukey’s multiple comparisons test. *P < 0.05 vs WT same time point, ^*#*^*P* < 0.05 vs baseline same genotype, ^§^*P* < 0.05 vs vehicle same genotype and time point.

In vehicle-treated WT and Npt2a^−/−^ mice, acute oral P_i_ loading resulted in a significant increase in plasma P_i_ levels (2.4 ± 0.1 and 3.1 ± 0.1 mmol L^−1^, respectively). In vitamin D_3_-treated WT and Npt2a^−/−^ mice, acute oral P_i_ loading resulted in a significantly greater increase in plasma P_i_ levels (3.9 ± 0.2 and 4.2 ± 0.3 mmol L^−1^, respectively) compared to their respective vehicle-treated genotype (Fig. 4).

### Vitamin D_3_ treatment causes significant Ca^2+^ accumulation in the kidney of Npt2a^−/−^ mice

**Figure 5:**
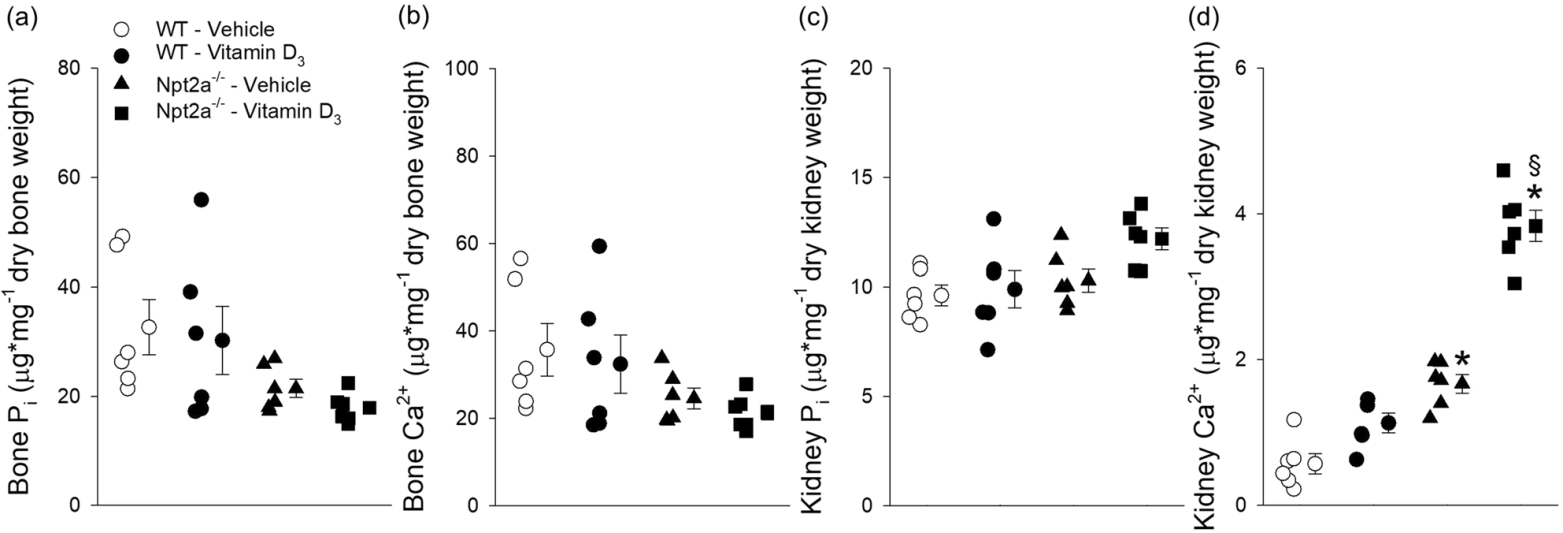
Vitamin D_3_ treatment increases kidney Ca^2+^ levels in Npt2a^−/−^ mice. Measurements of P_i_ and Ca^2+^ levels were carried out in bone and kidney tissues of WT and Npt2a^−/−^ mice following 4 days of treatment with either a vehicle or vitamin D_3_ (n = 6 per genotype). (**a** & **b**) There was no difference in the levels of P_i_ and Ca^2+^ in bone of either genotype in response to vehicle or vitamin D_3_ treatment. (**c**) Kidney P_i_ levels were not significantly different between genotypes or treatment. (**d**) The kidney Ca^2+^ levels were significantly greater in response to vehicle treatment in Npt2a^−/−^ compared to WT mice. Vitamin D_3_ treatment was not associated with altered kidney Ca^2+^ levels in WT mice; however, resulted in the highest kidney Ca^2+^ levels in Npt2a^−/−^ mice. Male mice were used in these studies. In addition to single data summary data are shown and are expressed as mean ± SEM and were analyzed by two-way ANOVA followed by Tukey’s multiple comparisons test. *P < 0.05 vs WT same treatment, ^§^*P* < 0.05 vs vehicle same genotype.

Determination of Ca^2+^ and P_i_ amounts were conducted on ashed tissue from vehicle and vitamin D_3_-treated WT and Npt2a^−/−^ mice. Amounts of P_i_ or Ca^2+^ in bone were not significantly different between genotypes in response to vehicle or vitamin D_3_ treatment (Fig. 5A,B). Similarly, kidney P_i_ levels were not significantly different between genotypes in response to vehicle or vitamin D_3_ treatment (Fig. 5c). Of note, kidney Ca^2+^ levels in response to vehicle treatment were significantly greater (~ threefold) in Npt2a^−/−^ compared to WT mice (Fig. 5d). Kidney Ca^2+^ levels were not significantly different between vehicle- and vitamin D_3_-treated WT mice. In contrast, vitamin D_3_ treatment in Npt2a^−/−^ mice resulted in the highest kidney Ca^2+^ content observed between groups and genotypes and was ~ 3.5-fold greater compared to vitamin D_3_-treated WT mice.

### Vitamin D_3_ treatment causes signs of impaired renal function associated with severe renal Ca^2+^-P_i_ deposits in Npt2a^−/−^ mice

**Figure 6:**
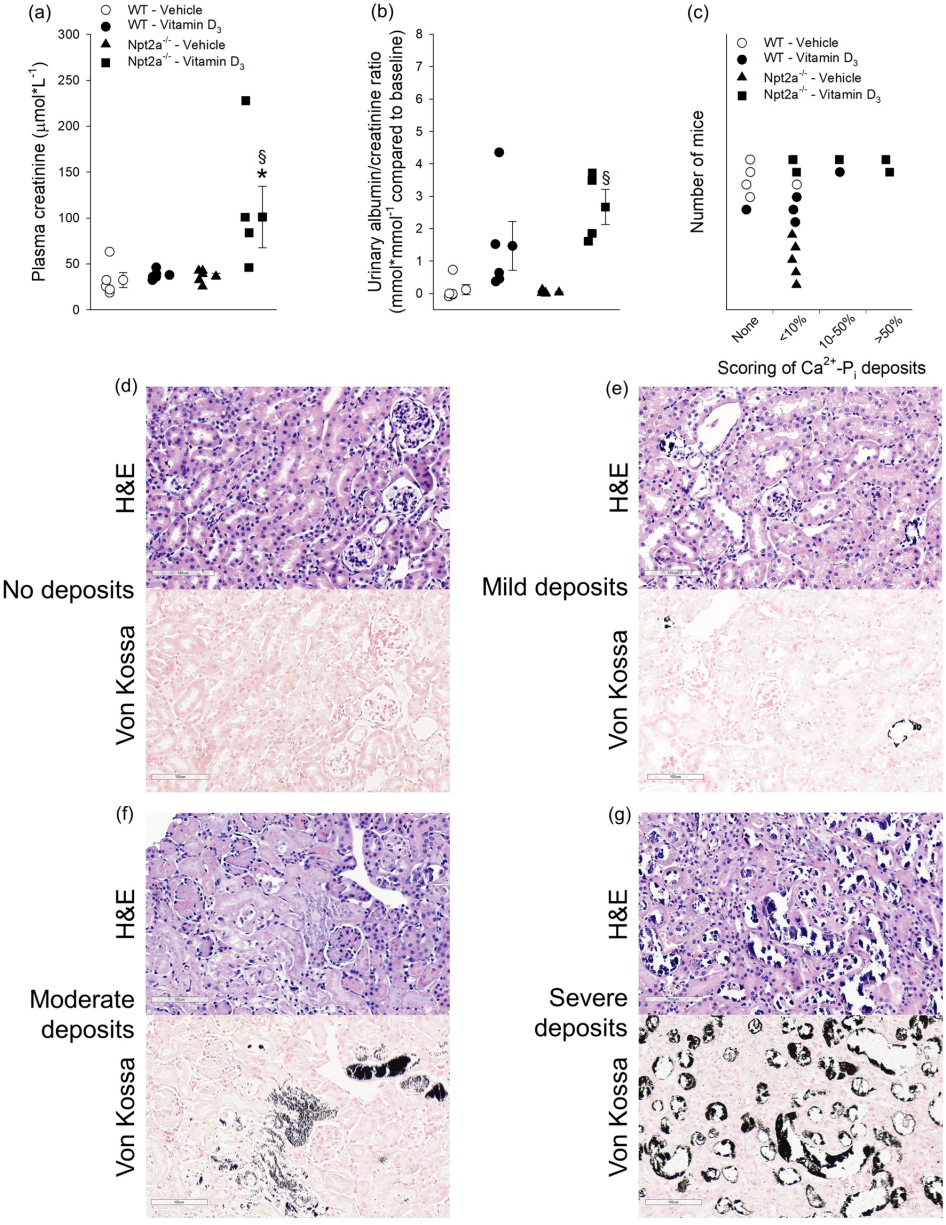
Vitamin D_3_-treated Npt2a^−/−^ mice show signs of impaired kidney function and greater renal Ca^2+^-P_i_ deposits. (**a**) Plasma creatinine levels were the highest in vitamin D_3_-treated Npt2a^−/−^ mice. (**b**) Similarly, urinary albumin/creatinine ratios showed the biggest increase compared to baseline in vitamin D_3_-treated Npt2a^−/−^ mice. (**c**) Histological classification of mineral deposits in genotypes with vehicle or vitamin D_3_ treatment. The majority of vehicle-treated WT mice show no mineral deposits, vehicle-treated Npt2a^−/−^ mice showed mild deposits (< 10%), vitamin D_3_-treated WT showed greater severity (moderate 10–50%) and only some vitamin D_3_-treated Npt2a^−/−^ mice showed the greatest number of deposits (> 50%). Representative examples of H&E and von Kossa staining are shown for each condition from mice with no mineral deposits (**d**), mild deposits (**e**), moderate deposits (**f**) and severe deposits (**g**). Von Kossa staining shows that Ca^2+^-P_i_ crystal deposits (black stains) localize within the tubular lumen. The tubules show evidence of damage with attenuation of the epithelial lining. Magnification × 200. Scale bar of 100 µm is shown in each image. Male and female mice were used in these studies. In addition to single data summary data are shown and are expressed as mean ± SEM and were analyzed by two-way ANOVA followed by the two-stage linear step-up procedure of Benjamini, Krieger, and Yekutieli. *P < 0.05 vs WT same treatment, ^§^*P* < 0.05 vs vehicle same genotype.

Plasma creatinine shows the highest levels in vitamin D_3_-treated Npt2a^−/−^ mice, no differences were observed between the other groups (Fig. 6a). Urinary albumin/creatinine ratios also showed significantly increased levels in vitamin D_3_-treated Npt2a^−/−^ mice compared to vehicle-treated Npt2a^−/−^ mice (Fig. 6b). To visualize Ca^2+^-P_i_ deposits, we used von Kossa staining and performed semi-quantitative analysis (Fig. 6c–g). Consistent with plasma creatinine levels, vitamin D_3_-treated Npt2a^−/−^ mice show the most severe amount (> 50%) of crystal deposits in the tubular lumen. The tubules show evidence of damage with attenuation of the epithelial lining. In contrast to vitamin D_3_-treated Npt2a^−/−^ mice, the majority of vitamin D_3_-treated WT mice show only mild (< 10%) crystal deposits similar to vehicle-treated Npt2a^−/−^ mice.

### Renal mRNA expression

RT-qPCR profiling of genes (all shown in Supplementary Fig. 2) expressed in the kidney confirmed that primers for *Slc34a1* (Npt2a) were specific for Npt2a since no mRNA amplification was found in Npt2a^−/−^ mice. Vehicle treatment showed significantly lower *CaSR* (Ca^2+^-sensing receptor) and *Cldn19* (claudin-19) expression in Npt2a^−/−^ compared with WT mice. In vitamin D_3_-treated WT mice, significant differences in mRNA expression were found compared to vehicle treatment for *Slc34a1*, *Slc34a3* (Npt2c), *Slc8a1* (Na^+^/Ca^2+^ exchanger, NCX1), *Atp2b4* (ATPase plasma membrane Ca^2+^ transporting 4), and *CaSR*. In vitamin D_3_-treated Npt2a^−/−^ mice, significant differences in mRNA expression were observed compared to vehicle treatment for *Slc34a3*, *Atp2b4*, *Trpv5* (transient receptor potential cation channel subfamily V member 5), *CaSR*, *Cyp27b1* (25-hydroxyvitamin D-1α-hydroxylase) and *Cldn16* (claudin-16). Significant differences between genotypes in response to vitamin D_3_ treatment were found for *CaSR*, *Cyp27b1*, and *Cldn16*.

### Npt2a maintains Npt2c and claudin-3 expression in response to vitamin D_3_

**Figure 7:**
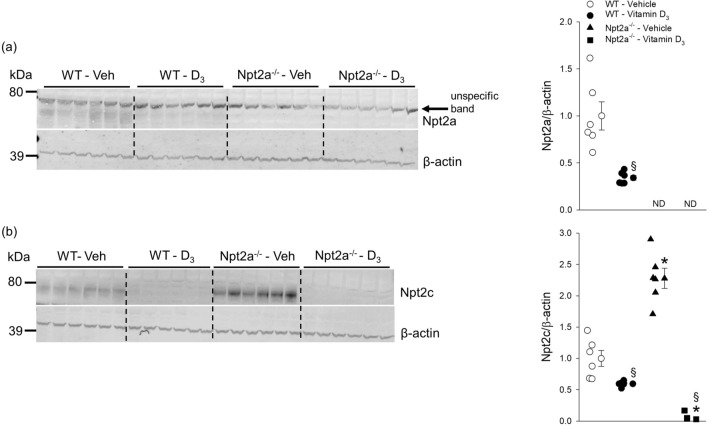
Npt2c abundance is diminished in Npt2a^−/−^ mice in response to vitamin D_3_ treatment. Abundance of Npt2a and Npt2c in kidney tissues of WT and Npt2a^−/−^ mice after 4 days of treatment with vehicle or vitamin D_3_ (n = 4–6 per genotype). (**a**) In this study we confirmed the specificity of the Npt2a antibody in Npt2a^−/−^ mice, which lack the ~ 75–80 kDa band representing Npt2a. An unspecific band was detected. In WT mice, vitamin D_3_ treatment showed lower Npt2a expression compared to vehicle treatment. (**b**) In response to vehicle treatment, Npt2c abundance was significantly greater in Npt2a^−/−^ compared to WT mice. Npt2c abundance was significantly lower in vitamin D_3_-treated mice; however, the level in Npt2a^−/−^ mice was almost undetectable. Male mice were used in these studies. In addition to single data summary data are shown and are expressed as mean ± SEM and were analyzed by two-way ANOVA followed by Tukey’s multiple comparisons test. *P < 0.05 vs WT same treatment, ^§^*P* < 0.05 vs vehicle same genotype.

**Figure 8:**
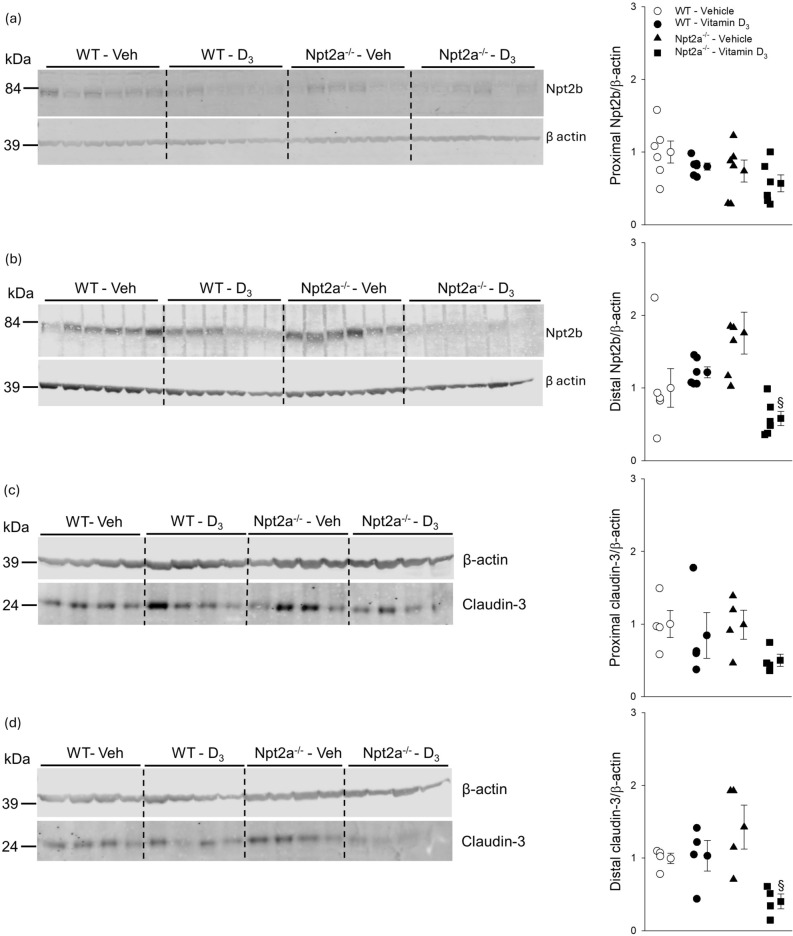
Npt2b abundance is diminished in Npt2a^−/−^ mice in response to vitamin D_3_ treatment. (**a**) Abundance of Npt2b in the proximal small intestine was not different between genotypes or treatment. (**b**) In the distal small intestine, no differences were detected in Npt2b abundance between vehicle-treated genotypes. In WT mice, Npt2b abundance was similar in response to vitamin D_3_ compared to vehicle treatment. Of note, Npt2b abundance in vitamin D_3_ -treated Npt2a^−/−^ mice was lower compared to vehicle treatment. (**c**) Abundance of claudin-3 was somewhat variable in the proximal small intestine and no differences were observed between genotype or treatment. (**d**) In the distal small intestine, no differences in claudin-3 abundance were detected between vehicle-treated genotypes. In WT mice, claudin-3 abundance was similar in response to vitamin D_3_ compared to vehicle treatment. Of note, claudin-3 abundance in vitamin D_3_-treated Npt2a^−/−^ mice was lower compared to vehicle treatment. Male mice were used in these studies. In addition to single data summary data are shown and are expressed as mean ± SEM and were analyzed by two-way ANOVA followed by Tukey’s multiple comparisons test. ^§^*P* < 0.05 vs vehicle same genotype.

Confirming the specificity of the Npt2a antibody, no Npt2a band was observed in kidney tissue of Npt2a^−/−^ mice (Fig. 7a). In response to vitamin D_3_ treatment in WT mice, Npt2a abundance was significantly lower (~ 65%) compared to vehicle-treated mice (Fig. 7a). Consistent with a compensatory response of Npt2c abundance in Npt2a^−/−^ mice, Npt2c abundance was ~ 3.5-fold greater in vehicle-treated Npt2a^−/−^ mice compared to vehicle-treated WT mice (Fig. 7b). In response to vitamin D_3_ treatment in WT mice, Npt2c abundance was significantly lower (~ 40%) compared to vehicle-treated WT mice. Of note, vitamin D_3_ treatment completely diminished Npt2c abundance in Npt2a^−/−^ mice. Since vitamin D_3_ affects intestinal P_i_ transport, we further analyzed intestinal abundance of Npt2b and claudin-3, the latter being a paracellular tight junction protein involved in P_i_ transport^21^. The majority of intestinal P_i_ uptake in the mouse occurs in the distal small intestine^22,23^. The abundance of Npt2b in the proximal small intestine was not significantly different between genotypes or treatments (Fig. 8a). No differences were observed in the abundance of Npt2b in the distal small intestine between genotypes in response to vehicle treatment. In WT mice (Fig. 8b), Npt2b abundance was not significantly different in response to vitamin D_3_ compared to vehicle treatment. In contrast, Npt2b expression in the distal small intestine of Npt2a^−/−^ mice was significantly lower (~ 77%) in response to vitamin D_3_ compared to vehicle treatment (Fig. 8b).

In the proximal small intestine of WT mice, claudin-3 protein abundance was not significantly different between vehicle or treatment groups (Fig. 8c). In the distal small intestine, no significant differences were observed in claudin-3 abundance between genotypes in response to vehicle treatment (Fig. 8d) and in WT mice no significant differences were observed between vehicle and vitamin D_3_ treatment. In contrast, in Npt2a^−/−^ mice, claudin-3 protein abundance was significantly lower (~ 72%) in response to vitamin D_3_ compared to vehicle treatment (Fig. 8d).

## Discussion

**Figure 9:**
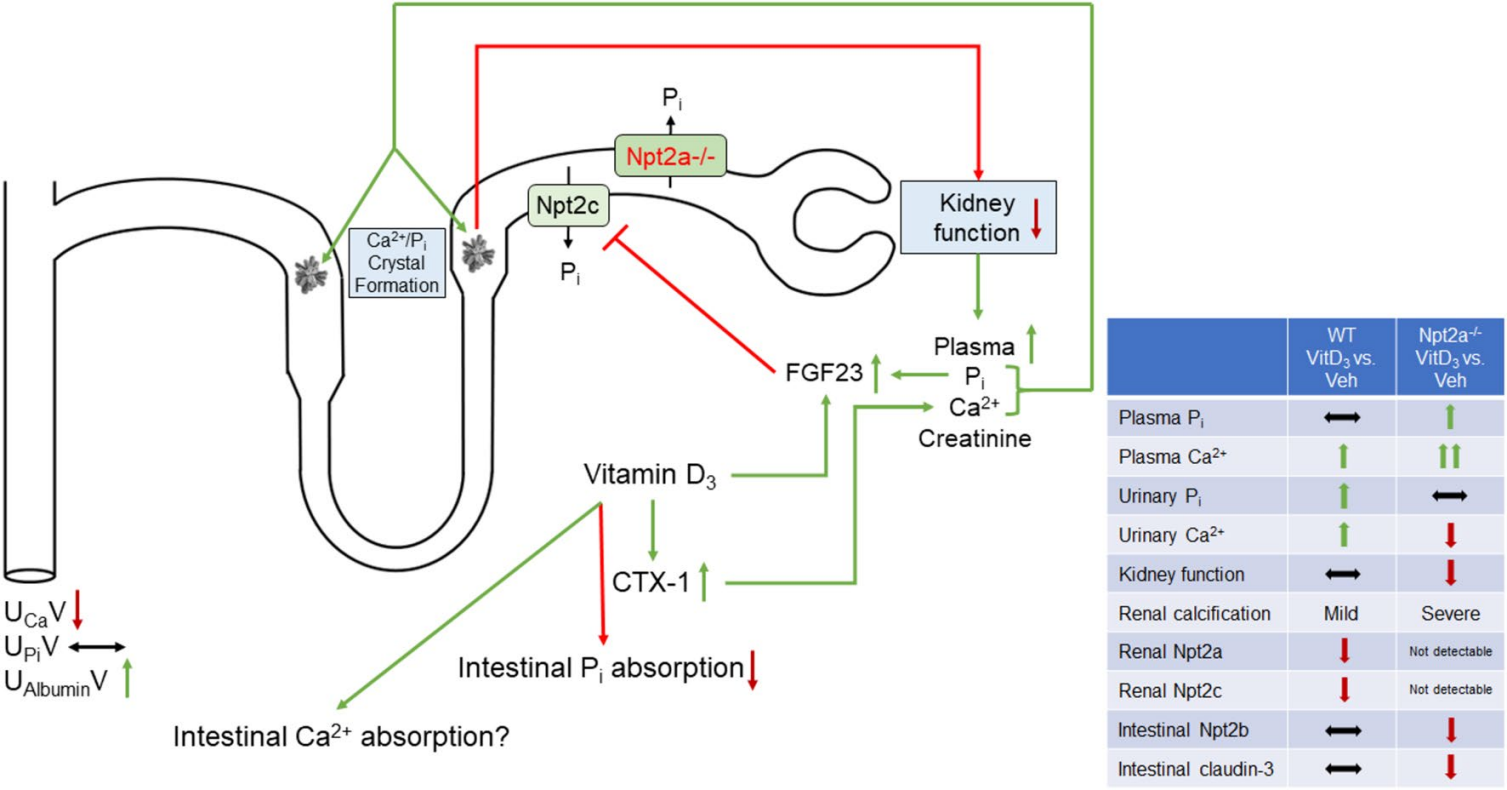
Summary figure. In vitamin D_3_-treated Npt2a^−/−^ mice, a circulus vitiosus is observed leading to kidney failure. We hypothesize that vitamin D_3_ treatment leads to elevated plasma Ca^2+^ levels (possibly via increased bone resorption as indicated by greater CTX-1 levels and/or increased intestinal Ca^2+^ absorption) and decreased intestinal P_i_ absorption (via lower Npt2b and claudin-3 levels). A combination of vitamin D_3_, elevated plasma P_i_, and reduced kidney function causes FGF23 levels to be drastically elevated, subsequently diminishing Npt2c abundance and leading to supersaturation of tubular fluid with Ca^2+^ and P_i_. Formation of Ca^2+^/P_i_ crystals lead to renal calcification and reduced kidney function (increase in plasma creatinine and urinary albumin). Consequently, plasma Ca^2+^ and P_i_ levels are further increased. Green arrows indicate an increase, red arrows indicate a decrease. The table on the right summarizes the most significant findings observed between vitamin D_3_ treated mice and vehicle treated mice for both genotypes.

The role of Npt2a in regulating renal P_i_ transport has been extensively studied. However, there are significant knowledge gaps when it comes to the complex hormonal regulation of this transporter, in particular the role of vitamin D_3_. To gain further mechanistic insight, we studied P_i_ and Ca^2+^ homeostasis when the body is challenged by exogenous administration of a high dose of vitamin D_3_ in the absence and presence of hyposphosphatemia, the latter caused by lack of Npt2a. Surprisingly, despite lack of Npt2a which should have facilitated renal P_i_ excretion, these mice show signs of impaired P_i_ excretion, possibly a results of greater nephrocalcinosis and a reduction of kidney function, in response to vitamin D_3_ administration (for a summary see Fig. 9).

Lack of Npt2a causes hypophosphatemia^16,18,20^, a finding confirmed in the current study. A similar situation can be induced by administration of a Npt2a inhibitor^20,24,25^. In WT mice, the administration of vitamin D_3_ did not affect plasma P_i_ levels; in contrast, Npt2a^−/−^ mice had a very uniform increase in plasma P_i_ following administration of vitamin D_3._ Regarding the former, other studies which administered vitamin D_3_ at a dose of 400,000 (IU kg^−1^) to C57Bl/6 mice did not report changes in plasma P_i_ levels^26^. It is conceivable that in WT mice, the increase in urinary P_i_/creatinine ratio served to stabilize plasma P_i_ levels. Consistent with this, vitamin D_3_ treatment resulted in lower Npt2a and Npt2c expression in WT mice.

Under baseline conditions, we did not find a clear P_i_ wasting phenotype in Npt2a^−/−^ mice. This could be related to a substantial compensatory greater Npt2c expression (seen on the protein but not mRNA level) which might mitigate the P_i_ wasting phenotype. It is notable that plasma P_i_ significantly increased in response to vitamin D_3_ treatment despite the absence of Npt2a. This corroborated with greater urinary albumin/creatinine ratios and greater plasma creatinine levels, possibly implying that reduced kidney function might have contributed to this finding. In addition, Npt2c abundance was also diminished in Npt2a^−/−^ mice under these conditions, both of which should facilitate urinary P_i_ excretion and prevent a rise in plasma P_i_. In terms of renal Npt2 transporter expression in response to vitamin D_3_, Npt2a^−/−^ mice resemble Npt2a/c double knockout mice. What could explain the lack of increase in urinary P_i_/creatinine ratios in response to vitamin D_3_ in Npt2a^−/−^ mice? One possible explanation could be that urinary P_i_ excretion has reached a maximum. Consistent with this, we have previously shown in short-term metabolic cage experiments that urinary P_i_/creatinine ratios in Npt2a^−/−^ mice were of the same magnitude, and only acute Npt2a inhibition in control mice was able to double urinary P_i_/creatinine^20^, suggesting that in Npt2a^−/−^ mice (chronically), despite the presence of compensatory mechanisms, no further increase in urinary P_i_ excretion can be achieved.

In terms of Ca^2+^, our data are consistent with the previously published Npt2a^−/−^ phenotype as well as the role of vitamin D_3_ in Ca^2+^ homeostasis^16,18^. Npt2a^−/−^ mice have higher plasma Ca^2+^ levels and greater urinary Ca^2+^/creatinine ratios. Vitamin D_3_ is a well-known regulator of intestinal Ca^2+^ absorption^27^ and knockout of Npt2a is associated with significantly increased intestinal Ca^2+^ absorption, possibly as a consequence of greater intestinal mRNA expression of epithelial Ca^2+^ channels (ECaC1 and ECaC2) and the Ca^2+^ binding protein calbindin-D9k^28^. Vitamin D_3_ treatment increased plasma Ca^2+^ in both genotypes, but to a greater amount in Npt2a^−/−^ mice. Of note, one of the most interesting findings in this study relates to the response of the kidney after administration of vitamin D_3_. In WT mice, urinary Ca^2+^/creatinine was appropriately increased possibly as a consequence of significant hypercalcemia. In contrast to WT mice, in Npt2a^−/−^ mice urinary Ca^2+^/creatinine was reduced in response to vitamin D_3_ administration, reaching levels seen in WT mice under baseline conditions. Consequently, the reduction of urinary Ca^2+^/creatinine ratio could have contributed to the greater increase in plasma Ca^2+^ in Npt2a^−/−^ mice. Despite vitamin D response elements being present in the CaSR gene causing up-regulation of CaSR expression^29^, our study provides evidence that vitamin D_3_ treatment can reduce CaSR expression.

Ultrastructural studies in Npt2a^−/−^ mice showed that at early age Ca^2+^/P_i_ deposits develop that were purged during later stages of life^30,31^. Along those lines, our tissue analysis showed that vitamin D_3_-treated Npt2a^−/−^ mice had the highest kidney Ca^2+^ content of all studied groups, without significant differences in kidney P_i_ content. Of note, Npt2a mutations in humans seem fairly common in a large cohort of Ca^2+^-stone forming pedigrees, but they do not seem to corroborate with clinically significant P_i_ or Ca^2+^ handling abnormalities^32^. Our studies expand this knowledge and show that vitamin D_3_-treated Npt2a^−/−^ mice show the greatest amount of Ca^2+^-P_i_ crystal deposits in the tubule lumen. Of note, vitamin D_3_-treated WT mice show a similar pattern of Ca^2+^-P_i_ crystal deposits compared to vehicle-treated Npt2a^−/−^ mice. Taken together, Npt2a^−/−^ mice have a significant problem in excreting Ca^2+^ in their urine and, considering also the lack of Npt2c in response to vitamin D_3_ administration, their kidney Ca^2+^ content is further consistent with the phenotype of Npt2a/c double knockout mice, which show severe renal calcifications^18^.

Possibly because of a combination of hypophosphatemia and hypercalcemia in Npt2a^−/−^ mice, PTH and FGF23 levels are significantly lower compared to WT mice^16–18^ which is still present when a high P_i_ or high P_i_/Ca^2+^ diet is provided^17^. Under baseline conditions, these findings were confirmed in our study. PTH synthesis and release under these conditions seems to be under a dual control: (1) hypercalcemia inhibits the synthesis and secretion of PTH from the parathyroid gland via activation of the CaSR and (2) active vitamin D_3_ suppresses the synthesis and release of PTH via activation of the VDR^33^. Of note, the changes observed cannot explain the paradoxical response of urinary Ca^2+^/creatinine in Npt2a^−/−^ mice in response to vitamin D_3_. The situation in vitamin D_3_-treated Npt2a^−/−^ mice is similar to hereditary hypophosphatemic rickets with hypercalciuria^34^ a physiology also resembled in Npt2a/c double knockout mice^18^. One notable difference is the accumulation of Ca^2+^ in the kidney of vitamin D_3_-treated Npt2a^−/−^ mice rather than the development of a hypercalciuric response. Along those lines, PTH was already substantially reduced in Npt2a^−/−^ mice under baseline conditions possibly in the face of lower plasma P_i_ and elevated plasma Ca^2+^ levels. The decrease of PTH in WT mice in response to vehicle treatment possibly relates to the presence of ethanol which has been shown to decrease PTH levels^35,36^.

Consistent with previous reports, our study confirms the lower FGF23 levels in Npt2a^−/−^ mice, a possible consequence of lower plasma P_i_ levels^18^. Our study did not determine 1,25(OH)_2_D_3_ levels but levels were found to be significantly greater in Npt2a^−/−^ compared with WT mice^16,18^. Our data show that vitamin D_3_ treatment does not affect *Cyp24a1* and *Cyp27b1* mRNA expression in WT mice. In chronic kidney disease, expression of *Cyp24a1* is increased possibly accounting for decreased 1,25(OH)_2_D_3_ due to degradation^37^. Vehicle treatment in Npt2a^−/−^ mice showed significantly lower expression compared to WT mice, possibly explaining the body’s effort to increase 1,25(OH)_2_D_3_ levels. Only in vitamin D_3_-treated Npt2a^−/−^ mice was *Cyp27b1* mRNA expression significantly increased, which is consistent with greater 1,25(OH)_2_D_3_ production. Study participants treated with vitamin D_3_ normally do not show increases in 1,25(OH)_2_D_3_ levels^38^, which is reflected in unchanged *Cyp27b1* levels in vitamin D_3_-treated WT mice compared to vehicle treatment. However, in the absence of Npt2a this can be offset, and our data imply that *Cyp27b1* mRNA expression is paradoxically increased.

When VDR are knocked out in chondrocytes of mice, FGF23 expression in osteoblasts and consequently FGF23 plasma levels are significantly reduced, implying that VDR is a prerequisite in this signaling pathway^39^. Of note, exogenous administration of vitamin D_3_ is a powerful stimulator of FGF23, leading to ~ 80–200-fold increase; however, the exact signaling pathway(s) causing this increase remain unclear and the relationship between these hormones is complex. Our results dispute the role of PTH being a major determining factor for FGF23 production (which was drastically suppressed in both genotypes), or suggest additional regulatory mechanisms, which has been demonstrated in vivo and in vitro as well as in mice with hyperparathyroidism^40,41^. Vice versa, our data are consistent with the notion that FGF23 reduces PTH synthesis directly^42^.

Npt2a^−/−^ mice show a skeletal phenotype characterized by delayed secondary ossifications at 21 days of age which are reversed at 45 days of age and are ultimately overcompensated at > 74 days of age^16^, these effects are even more exaggerated in Npt2a/c double knockout mice^18^. Our study used highly sensitive bone remodeling markers as estimators, which, to our knowledge, have never been determined in Npt2a^−/−^ mice. Despite significant differences in P_i_ and Ca^2+^ homeostasis between genotypes, our study did not identify changes in any of the bone remodeling markers studied under baseline conditions. This might relate to the fact that Npt2a^−/−^ mice were of adult age when our studies were performed. Osteocalcin is predominantly produced and secreted by osteoblasts during bone formation^43^. Although low doses of vitamin D_3_ can stimulate bone turnover, high doses can cause bone resorption^44^. Despite these results, our study did not provide any differences between treatment or genotype in terms of osteocalcin levels. A possible explanation could be the short-term experimental setup we employed. PINP is considered the most sensitive marker of bone formation^45^, which has been reported to be under the control of PTH^46^ and shows an inverse relationship with active vitamin D_3_^47^. Consistent with this, both genotypes decreased PINP levels after vitamin D_3_ administration, consistent with a role of reduced bone formation. Of note, this occurred despite a significant decrease of PTH in both genotypes.

TRAcP 5b is an osteoclast-derived marker of bone resorption.^48^. Our findings show that vitamin D_3_ treatment increased TRAcP 5b in WT mice. So far, no correlations have been described between FGF23 and TRAcP 5b under normal conditions; however, in patients on evocalcet treatment (CaSR agonist) PTH, FGF23 and TRAcP 5b decreased over the 30 week treatment period^49^. Our data point toward a role of Npt2a in this process since Npt2a^−/−^ mice lack a response in TRAcP 5b in response to vitamin D_3_. CTX-1 is a marker for bone remodeling that is released when type 1 collagen is degraded^50^. The role of vitamin D_3_ on CTX-1 is ambiguous, with several human studies showing no effect of vitamin D_3_ supplementation on CTX-1 levels^51,52^ whereas others show a positive correlation^47^. This might relate to the pre-existing conditions that were studied, e.g. presence or absence of vitamin D_3_ deficiency. Of note, and consistent with our study, a study in humans showed a dose-dependent effect of vitamin D_3_ bolus administration (up to 600,000 IU) on CTX-1 levels 1 day after administration^53^. This might explain an increase in fracture risk when elderly women are treated annually with a single high dose (500,000 IU) of vitamin D_3_^54^. In addition, daily doses of 10,000 IU for 3 years also resulted in a significant increase of CTX-1 in healthy adults^44^. Lack of Npt2a possibly unravels that these mice are more susceptible for disturbed bone remodeling.

The intestine plays a vital role in P_i_ and Ca^2+^ absorption in order to regulate homeostasis in the body and vitamin D_3_ has been implicated in this regulation^14,28^. Our acute P_i_ loading experiments confirm these findings: independent of genotype, the intestinal uptake of P_i_ was significantly greater in vitamin D_3_-treated mice compared to vehicle-treated mice as evidenced by greater increases in plasma P_i_ levels in the face of reduced renal Npt2a/c abundance. Our studies on Npt2b abundance also expand the knowledge on spatial regulation, where abundance in response to Npt2b was unaffected in the proximal small intestine, which contrasts with the distal small intestine. Of note, the contribution of transcellular versus paracellular intestinal P_i_ transport is a highly debated topic^15,23^, and claudin-3 has been implicated in the paracellular process. Supporting this hypothesis are data from claudin-3 knockout mice, which have enhanced intestinal P_i_ uptake^21^. Similar to Npt2b, no regulation of claudin-3 abundance in the proximal small intestine was found in our studies, but in the distal small intestine of Npt2a^−/−^ mice, claudin-3 abundance was significantly reduced, possibly contributing to greater plasma P_i_ levels in Npt2a^−/−^ mice. In the kidney we find evidence that claudin-16, expressed in the thick ascending limb and distal convoluted tubule, was significantly reduced in vitamin D_3_-treated Npt2a^−/−^ mice. Claudin-16 inactivating mutations in humans are associated with hypercalciuria and nephrocalcinosis^55,56^, possibly suggesting that significantly reduced claudin-16 expression in our studies might have contributed to the phenotype of vitamin D_3_-treated Npt2a^−/−^ mice. Along those lines, claudin-16 interacts with Trpv5 since knockdown of claudin-16 delocalized Trpv5 from the luminal membrane^57^. In our study, Trpv5 was also significantly reduced in vitamin D_3_-treated Npt2a^−/−^ mice compared to vehicle-treated Npt2a^−/−^ mice. Our data provide information on the regulation of Atp2b4, which was reduced in response to vitamin D_3_ in both genotypes; however, knockout of Atp2b4 in mice did not cause a Ca^2+^ phenotype^58^.

In summary, our data provide novel insight into the role of vitamin D_3_ in the regulation of P_i_ and Ca^2+^ homeostasis in the context of Npt2a. One limitation of using mice to study P_i_ homeostasis relates to distinct differences in intestinal and renal P_i_ handling compared to humans. Despite the vitamin D_3_ dose used in our studies is supraphysiological, significant differences were observed between genotypes that pinpoint to an important role of Npt2a (and possibly claudin-16) in renal calcification and consequently kidney function decline. It is noteworthy that vitamin D_3_ treatment in Npt2a^−/−^ mice resulted in a complete loss of Npt2c, and mice in terms of renal P_i_ transporter expression resembled Npt2a/c double knockout mice. Despite a complete lack of renal P_i_ transporters, Npt2a^−/−^ mice experience greater plasma P_i_ levels, possibly a consequence of reduced intestinal claudin-3 abundance. Further, Npt2a^−/−^ mice develop significantly greater plasma Ca^2+^ levels in response to vitamin D_3_, possibly a consequence of impaired renal Ca^2+^ excretion with tissue accumulation of Ca^2+^, implying that Npt2a can function as a switch between renal Ca^2+^ excretion and reabsorption. However, the contribution of Npt2c in this process cannot be excluded considering its absence in abundance in response to vitamin D_3_ treatment in Npt2a^−/−^ mice.

## Methods

The animal experiments were conducted in compliance with the NIH Guide for Care and Use of Laboratory Animals, set by the National Institutes of Health (Bethesda, MD), received approval from the Institutional Animal Care and Use Committee (11201R) at the University of South Florida, and are reported in accordance with ARRIVE guidelines. Npt2a^−/−^ mice were obtained from the Jackson Laboratory (strain# 004802, Bar Harbor, ME) and propagated by heterozygote breeding. Mice have been backcrossed to C57BL/6J for 9 generations. Only male WT and Npt2a^−/−^ mice, 3–5 months old, were used for the study. The specific pathogen free mice were group housed and kept in a controlled environment with a 12-h light–dark cycle (light off at 18:00) in isolated ventilated cages. They were provided with free access to standard rodent diet (TD.2018, containing 0.7% P_i_ and 1% Ca^2+^, Envigo, Madison, WI) and drinking water. Genotype was determined by PCR amplification of genomic DNA, which was extracted from ear tissue samples. The genotyping was carried out in accordance with protocol # 29530 published on the Jackson Laboratory website.

### Vitamin D_3_ treatment

Wild-type and Npt2a^−/−^ mice were randomized into two treatment groups: one vehicle (5% Ethanol, 5% Cremophor EL, and 90% water) or vitamin D_3_ (3,000 and 300,000 IU/kg body weight, Alfa Aesar, Haverhill, MA) dissolved in vehicle^26^. Treatments were administered on 4 consecutive days via subcutaneous injections (2 µL g^−1^ body weight) by and investigator blinded to genotype and treatment. Blood samples were collected under brief isoflurane anesthesia from the retrobulbar plexus before and after the 4-day treatment period. Spontaneously voided urine was collected at the same time.

### Analysis of plasma and urine samples

Clinical chemistry was performed utilizing commercially available assays, adapted for use with small sample volumes^20,23^. Concentrations of P_i_ and Ca^2+^ in both plasma and urine were measured using inorganic phosphorous reagent and calcium arsenazo III reagent respectively, (Pointe Scientific, Canton, MI)^59^. Urinary creatinine was measured by infinity creatinine liquid stable reagent (Thermo Fisher Scientific, Middletown, VA). Urinary albumin and plasma creatinine were determined as described previously^60,61^. PTH and intact FGF23 were measured according to the manufacturer instructions (Quidel, San Diego, CA). Markers for bone resorption (tartrate-resistant acid phosphatase isoform 5b [TRAcP 5b, Quidel] and type I collagen cross-linked C-telopeptide [CTX-1, Immunodiagnostic Systems]) and bone formation (procollagen type I N-propeptide [PINP, Immunodiagnostic Systems, Gaithersburg, MD] and osteocalcin [Quidel]) were measured using ELISAs.

### Acute hyperphosphatemic model

Four days after the administration of either vehicle or vitamin D_3_, WT and Npt2a^−/−^ mice were subjected to gavage of 0.5 mol L^−1^ NaH_2_PO_4_, 1% of body weight by an investigator blinded to genotype^23,62^. Before gavage and 60 min after administration, blood samples were collected under brief isoflurane anesthesia. Plasma P_i_ was measured as described above.

### Determination of Ca^2+^ and P_i_ content in the kidney

In another set of WT and Npt2a^−/−^ mice, femurs and kidneys were harvested under terminal isoflurane anesthesia 4 days after the last administration of vehicle or vitamin D_3_. The collected tissues were dried for 24 h at 50 °C. Following the drying process, the weight of each tissue was determined. Next, the tissues were incinerated at a temperature of 560 °C for 12 h in a muffle furnace (Thermolyne F48015-60, Thermo Fisher Scientific). The ashes from the incineration were dissolved in 0.75 mol L^−1^ HCl. Concentrations of Ca^2+^ and P_i_ in the dissolved samples were determined as described above.

### Histological analysis of kidneys

In a separate cohort of mice kidneys were perfused in vivo through the left ventricle with 4% PFA in phosphate buffered saline under isoflurane anesthesia. After kidneys were removed, they were fixed overnight in the same solution and subsequently paraffin embedded and sectioned at 4–6 μm. After deparaffinization and rehydration, sections were stained with hematoxylin and eosin (H&E) and von Kossa (to determine mineral deposits). Sectioning and staining were performed by Reliance Pathology Partners, LLC (Tampa, FL). Quantification of Ca^2+^-P_i_ deposits were performed using the following scheme: none, mild (< 10%), moderate (10–50%), or severe (> 50%). The highest score seen in sections was reported for each mouse. All scoring was performed by a pathologist (M.T.) blinded to sample identity.

### Isolation of intestinal epithelial cells

Another cohort of mice was randomized to administration of either vehicle or vitamin D_3_ as described above. Following the 4-day treatment, mice were anesthetized by isoflurane and their kidneys and small intestines removed. Isolation of intestinal epithelial cells (IEC) via Ca^2+^ chelation was performed as described previously^23,63,64^. The IEC pellets were prepared for immunoblotting as described below.

### Western blotting

Collected IEC and kidneys were homogenized in a buffer composed of 250 mmol L^−1^ sucrose and 10 mmol L^−1^ triethanolamine (Sigma-Aldrich, St. Louis, MO) containing Halt protease inhibitor cocktail and Halt phosphatase inhibitor cocktail (both Thermo Fisher Scientific). The homogenate was then subjected to a centrifugation process at 1000×*g* for 15 min followed by generation of plasma membrane-enriched samples (by centrifugation of the supernatant at 17,000×*g*) for 30 min. The pellets that emerged from this process were then resuspended and prepared for Western blotting. Protein quantity was determined using a bicinchoninic acid assay (Thermo Fisher Scientific). Samples of equal concentration were made by the addition of Laemmli sample buffer (final concentration of 0.1 mol L^−1^ SDS and 15 mg L^−1^ DTT). Samples were heated at 65 ^0^C for 15 min before immunoblotting. The samples were resolved on either NuPAGE 4–12% or 12% Bis–Tris gels in MOPS. Proteins were transferred to polyvinylidene difluoride membranes and immunoblotted with rabbit polyclonal antibodies against Npt2a, Npt2b, Npt2c (each with a dilution of 1:1500, generous gift from M. Levi)^23,24,62^, rabbit anti claudin-3 (dilution 1:1000, also rabbit-sourced, Thermo Fisher Scientific)^23^, and mouse anti β-actin (dilution 1:30,000, Sigma-Aldrich). These targets were then detected with secondary antibodies designed for rabbit (IRDye® 800CW donkey anti-rabbit IgG, at a dilution of 1:5000) or mouse (IRDye® 680RD donkey anti-mouse IgG, also at a dilution of 1:5000), using an Odyssey® CLx detection system (LI-COR Biosciences, Lincoln, NE). Quantification of the band intensities was carried out using Image Studio Lite for densitometric analysis (LI-COR Biosciences).

### Quantitative polymerase chain reaction from kidney and bone

Total RNA from kidney homogenates was extracted using Tri Reagent (Sigma-Aldrich) using a protocol adapted from the manufacturer’s recommendations. Total RNA was quantified using a Synergy Neo2 plate reader (Agilent, Santa Clara, CA). One thousand ng RNA of kidney sample were used to produce cDNA using a Revert Aid First Strand cDNA Synthesis Kit (Thermo Fisher Scientific). Maxima SYBR Green/ROX qPCR Master Mix (Thermo Fisher Scientific) was used in conjunction with a QuantStudio 6 Pro (Applied Biosystems, Thermo Fisher Scientific) for amplification. Template concentration was 1 ng µl^−1^ cDNA per 10 µl reaction (performed in triplicate) and used in conjunction with primer pairs specific for *Slc8a1, Slc34a1, Slc34a3, Trpv5, Atp2b4, Cyp25a1, Cyp27b1, Cldn2, Cldn14, Cldn16, Cldn19,* and *CaSR* with actin used as a reference gene (all primer sequences are provided in the Supplementary Information). Data analysis used the ^ΔΔ^Ct method, i.e. cycle thresholds (Ct), were normalized to actin expression, and compared with control.

### Statistical analyses

Data are expressed as mean ± S.E.M. Two-way ANOVA or repeated-measures two-way ANOVA followed by Tukey’s multiple comparison tests, or two-way mixed-effects ANOVA followed by the two-stage linear step-up procedure of Benjamini, Krieger, and Yekutieli, as indicated in the figure legends, were used to test for significant differences between genotype and/or treatment. All data were analyzed via GraphPad Prism (Version 10.1, Boston, MA) or SigmaPlot (Version 14, San Jose, CA, USA). Significance was considered at *P* < 0.05.

### Supplementary Information

Supplementary Information.

## Data Availability

The data that support the findings of this study are available from the authors upon reasonable request.
