# Supplementary material for: Vitamin D3 suppresses Npt2c abundance and differentially modulates phosphate and calcium homeostasis in Npt2a knockout mice
Source: Sci Rep. 2024 Jul 23;14:16997. doi: 10.1038/s41598-024-67839-4 (PMC11266651; doi:10.1038/s41598-024-67839-4)
Supplement: Supplementary file 1 — Supplementary Information. [file 41598_2024_67839_MOESM1_ESM.pdf]

Vitamin D_3 Suppresses Npt2c Abundance and Differentially Modulates Phosphate and Calcium Homeostasis in Npt2a Knockout Mice

Linto Thomas, Lashodya V. Dissanayake, Maryam Tahmasbi, Alexander Staruschenko, Sima Al Masri, Jessica A. Dominguez Rieg, Timo Rieg

Supplemental Table 1

Primer sequences used for mRNA expression.

Oligonucleotide	5′-sequence-3′
Slc8a1 forward	AGAGCTCGAATTCCAGAACGATG
Slc8a1 reverse	TTGGTTCCTCAAGCACAAGGGAG
Slc34a1 forward	TGATCACCAGCATTGCCG
Slc34a1 reverse	GTGTTTGCAAGGCTGCCG
Slc34a3 forward	TCACCATACATGCAGAGCTAGGAT
Slc34a3 reverse	CAGAGTAGGGTTGGGGACCTG
Trpv5 forward	TCCTTTGTCCAGGACTACATCCCT
Trpv5 reverse	TCAAATGTCCCAGGGTGTTTCG
Atp2b4 forward	CTTAATGGACCTGCGAAAGC
Atp2b4 reverse	ATCTGCAGGGTTCCCAGATA
Cyp24a1 forward	GAAGATGTGAGGAATATGCCCTATTT
Cyp24a1 reverse	CCGAGTTGTGAATGGCACACT
Cyp27b1 forward	ATGTTTGCCTTTGCCCAGA
Cyp27b1 reverse	GACGGCATATCCTCCTCAGG
Cldn2 forward	AAGGTGCTGCTGAGG GTA GA
Cldn2 reverse	AGTGGCAGAGATGGG ATT TG
Cldn14 forward	ACCCTGCTCTGCTTATCC
Cldn14 reverse	GCACGGTTGTCCTTGTAG
Cldn16 forward	CAAACGCTTTTGATGGGATTC
Cldn16 reverse	TTTGTGGGTCATCAGGTAGG
Cldn19 forward	CGGGCAGGTGCAATGCAAAC
Cldn19 reverse	CAGGAGACAGCAGTCAAAGTA
CaSR forward	CATCTGTTCCATCTGCATCC
CaSR reverse	TACAGAGACAGCTCGTTGGG

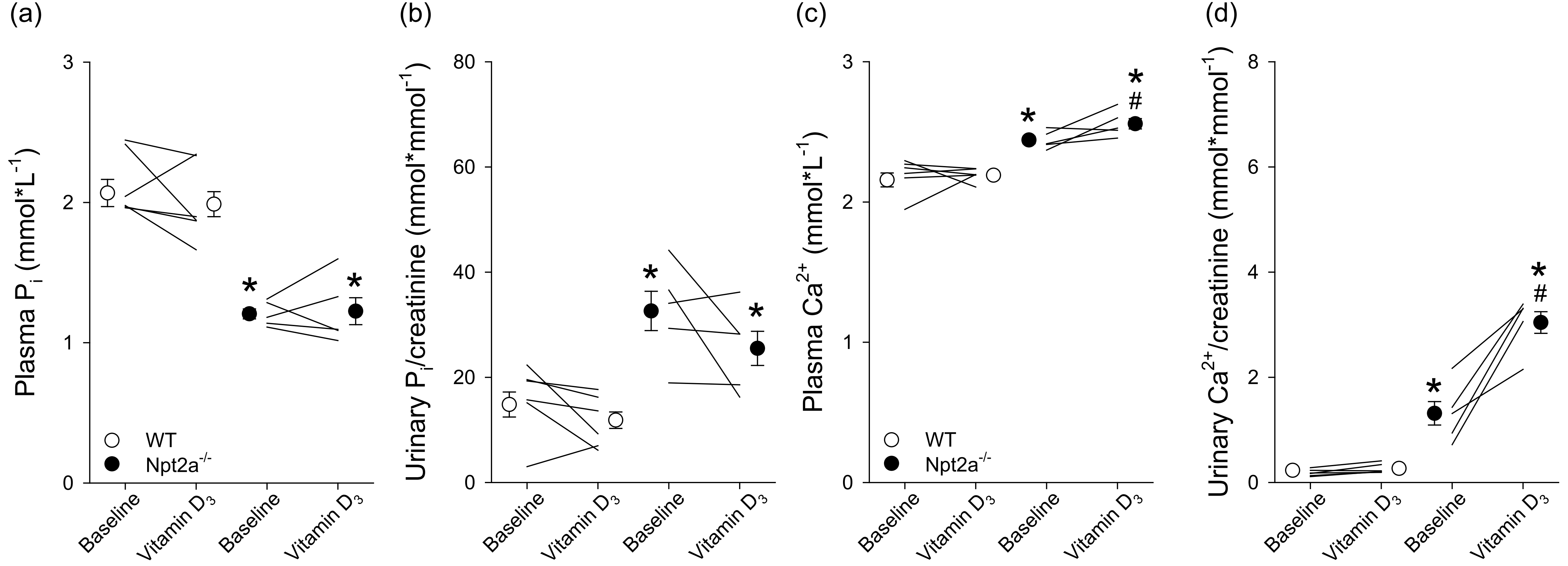

Supplementary Figure 1. Response of WT an Npt2a$^{-/-}$ mice to vitamin D_3 (3000 IU/kg body weight). Measurements of plasma and urinary P_i and Ca^{2+} were conducted in WT and Npt2a$^{-/-}$ mice after 4 days of treatment with vitamin D_3 (n=5-7 per genotype). (A) Plasma P_i levels were lower in Npt2a$^{-/-}$ mice and in both genotypes plasma P_i levels remained unchanged following vitamin D_3 treatment. (B) Urinary P_i/creatinine ratio was higher in Npt2a$^{-/-}$ mice but unchanged in both genotypes in response to vitamin D_3 treatment. (C) Plasma Ca^{2+} levels significantly increased following vitamin D_3 treatment in Npt2a$^{-/-}$ mice only. (D) In WT mice, the urinary Ca^{2+}/creatinine ratio was not changed after vitamin D_3 treatment. In contrast, in Npt2a$^{-/-}$ mice urinary Ca^{2+}/creatinine ratio significantly increased in response to vitamin D_3 treatment. Male and female mice were used in these studies. In addition to single data summary data are shown and are expressed as mean ± SEM and were analyzed by two-way ANOVA followed by the two-stage linear step-up procedure of Benjamini, Krieger, and Yekutieli. *P <0.05 vs WT same time point, $^{\#}P$ <0.05 vs baseline same genotype.

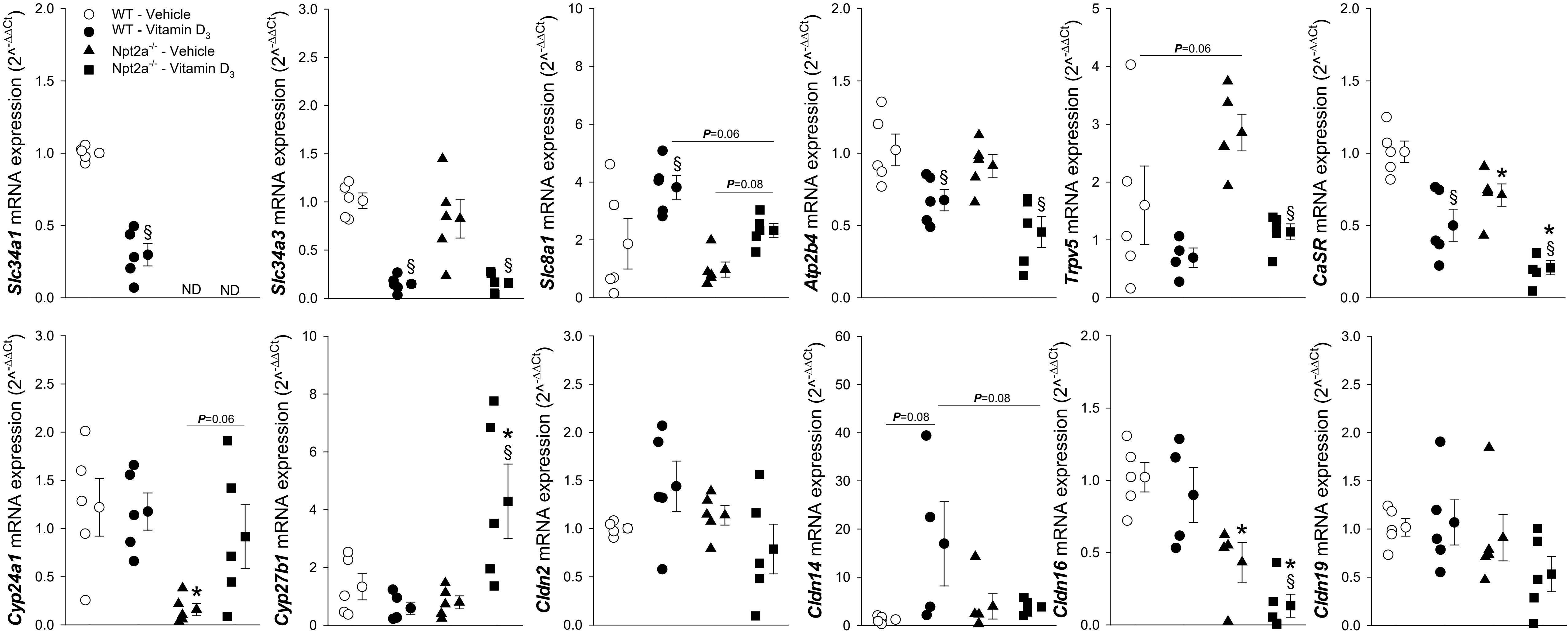

Supplementary Figure 2. Messenger RNA expression of different genes in the kidney. Top row from left to right: ***Slc34a1*** (Npt2a), ***Slc34a3*** (Npt2c), ***Slc8a1*** (NCX1), ***Atp2b4*** (ATPase plasma membrane Ca^{2+} transporting 4), ***Trpv5*** (transient receptor potential cation channel subfamily V member 5) and ***CaSR*** (Ca^{2+}-sensing receptor). Bottom row from left to right: ***Cyp24a1*** (25-Hydroxyvitamin D-24-hydroxylase), ***Cyp27b1*** (25-hydroxyvitamin D-1α-hydroxylase), ***Cldn2*** (claudin-2), ***Cldn14*** (claudin-14), ***Cldn16*** (claudin-16) and ***Cldn19*** (claudin-19). Male and female mice were used in these studies. In addition to single data summary data are shown and are expressed as mean ± SEM and were analyzed by two-way ANOVA followed by the two-stage linear step-up procedure of Benjamini, Krieger, and Yekutieli. ***P** <0.05 vs WT same treatment, §***P*** <0.05 vs vehicle same genotype.

(A)

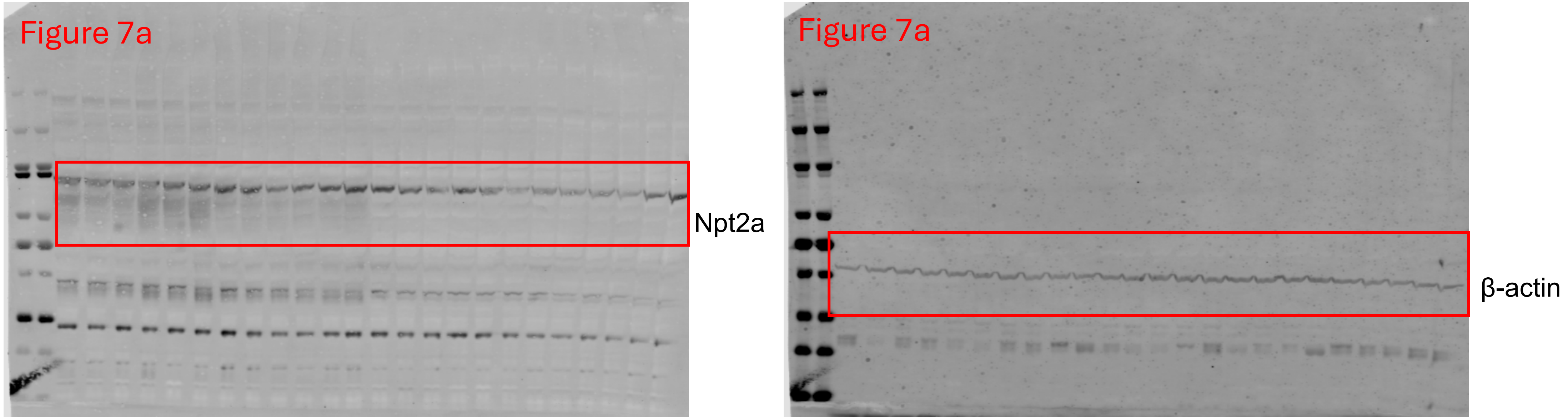
Figure 7a
Npt2a
Figure 7a
β-actin

(B)

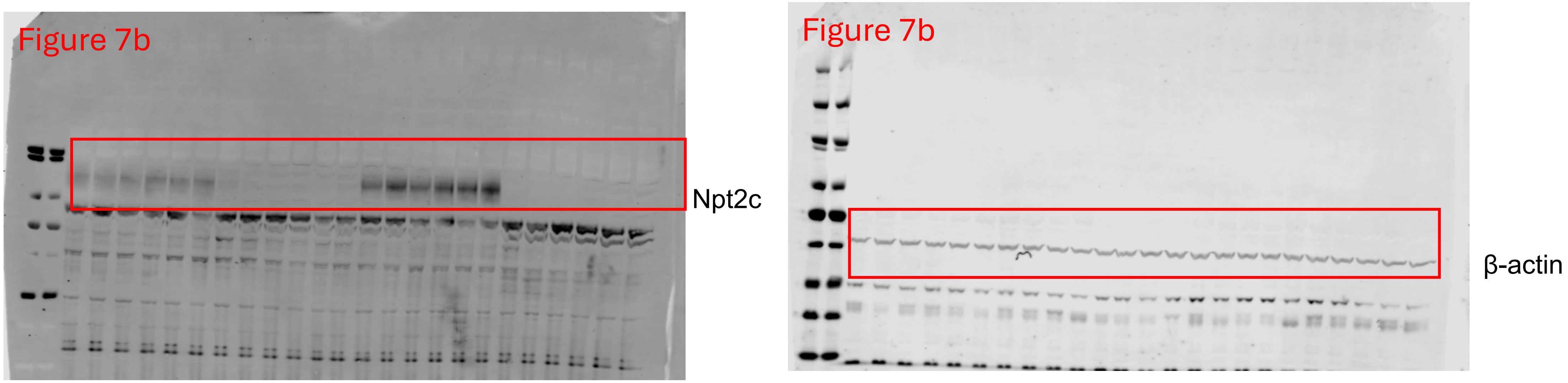
Figure 7b
Npt2c
Figure 7b
β-actin

(C)

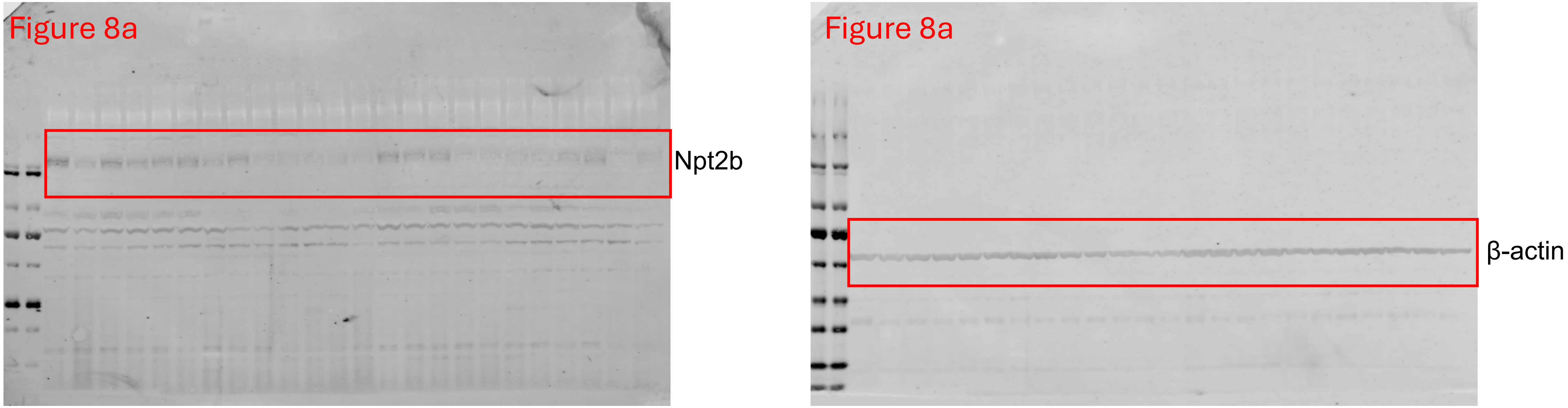
Figure 8a
Npt2b
Figure 8a
β-actin

(D)

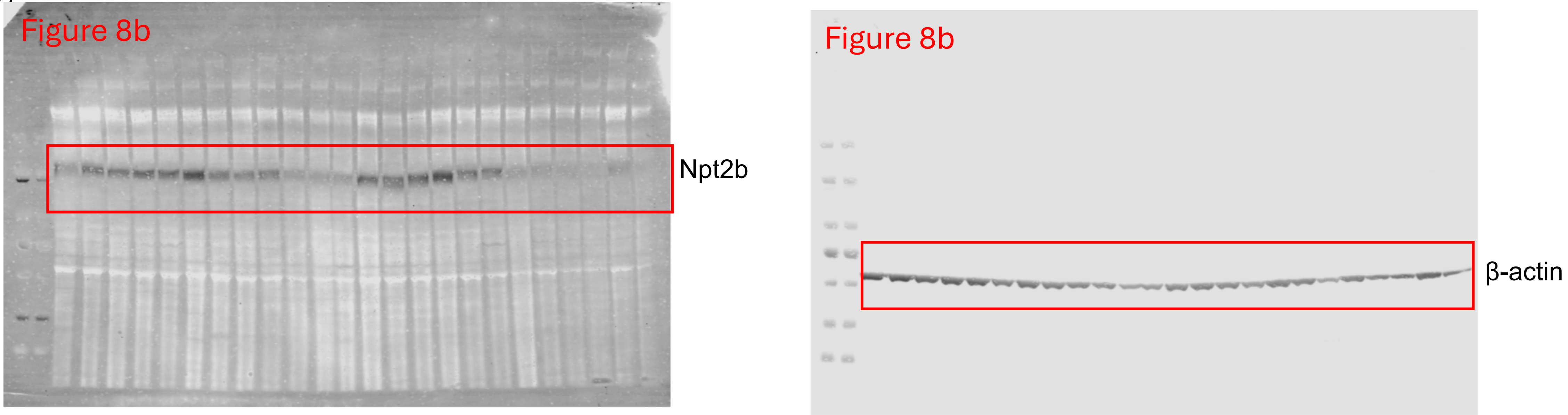
Figure 8b
Npt2b
Figure 8b
β-actin

(E)

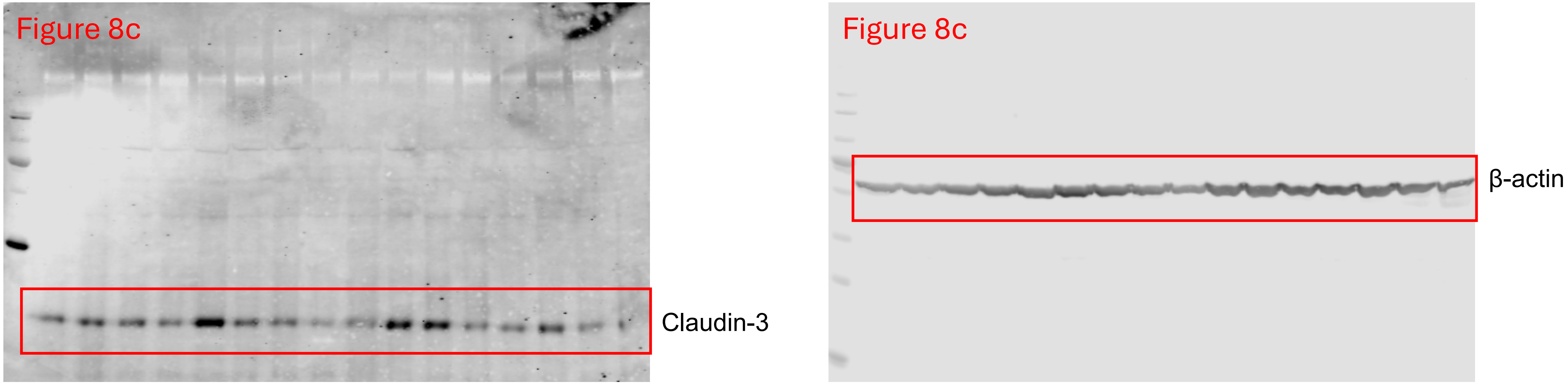
Figure 8c
Claudin-3
Figure 8c
β-actin

(F)

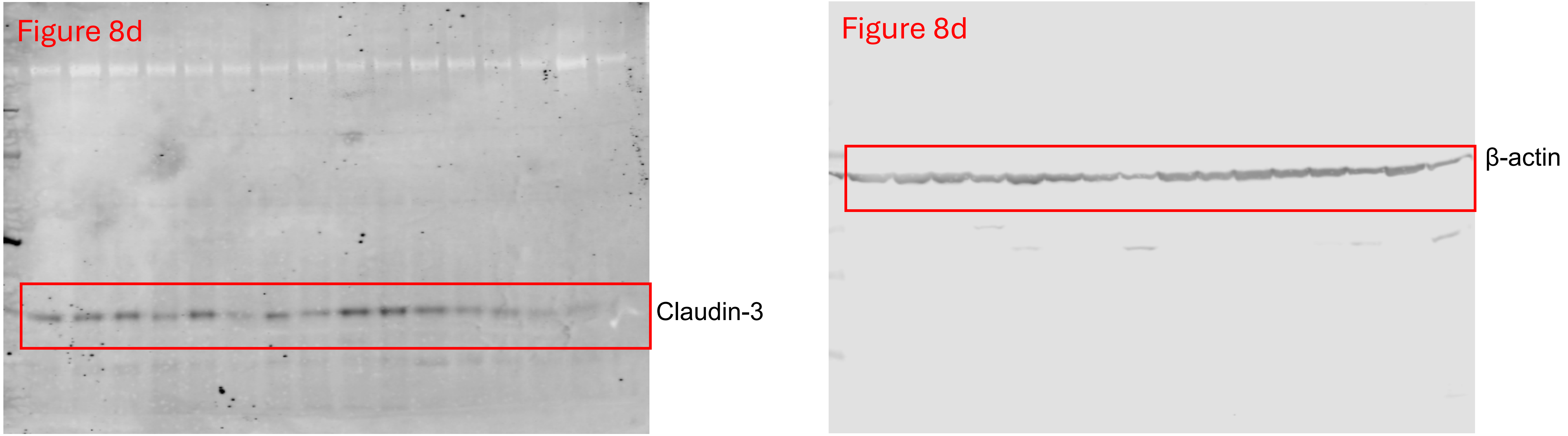
Figure 8d
Claudin-3
Figure 8d
β-actin
